# Transcriptome Analysis of *Gerbera* hybrida Including *in silico* Confirmation of Defense Genes Found

**DOI:** 10.3389/fpls.2016.00247

**Published:** 2016-03-01

**Authors:** Yiqian Fu, G. Danny Esselink, Richard G. F. Visser, Jaap M. van Tuyl, Paul Arens

**Affiliations:** Plant Breeding, Wageningen University and Research Centre (Wageningen UR)Wageningen, Netherlands

**Keywords:** EST, candidate gene, annotation, disease pathway, gerbera gray mold

## Abstract

For the ornamental crop *Gerbera* hybrida, breeding at the moment is done using conventional methods. As this has drawbacks in breeding speed and efficiency, especially for complex traits like disease resistance, we set out to develop genomic resources. The leaf and flower bud transcriptomes of four parents, used to generate two gerbera populations, were sequenced using Illumina paired-end sequencing. In total, 36,770 contigs with an average length of 1397 bp were generated and these have been the starting point for SNP identification and annotation. The consensus contig sequences were used to map reads of individual parents, to identify genotype specific SNPs, and to assess the presence of common SNPs between genotypes. Comparison with the non-redundant protein database (nr) showed that 29,146 contigs gave BLAST hits. Of sequences with blast results, 73.3% obtained a clear gene ontology (GO) annotation. EST contigs coding for enzymes were found in Kyoto Encyclopedia of Genes and Genomes maps (KEGG). Through, these annotated data and KEGG molecular interaction network, transcripts associated with the phenylpropanoid metabolism, other secondary metabolite biosynthesis pathways, phytohormone biosynthesis and signal transduction were analyzed in more detail. Identifying genes involved in these processes could provide genetic and genomic resources for studying the mechanism of disease resistance in gerbera.

## Introduction

*Gerbera* hybrida (2n = 2x = 50) is one of the most important ornamental plants and belongs to the Compositae family. Cultivated gerbera, which probably originates from a crossing of two wild species from Africa (*G. jamesonii* and *G. viridifolia*; Hansen, 1999), is highly heterozygous. Commercial gerbera cultivars are mainly produced in greenhouses for year round cut flower production (Moyer and Peres, 2008; Simpson, 2009) and ranked fifth in the cut flower sale at the Dutch flower auctions 2014 (https://www.floraholland.com/media/3949227/Kengetallen-2014-Engels.pdf). Gerbera became a model plant to study flower development in composed (Compositea) flowers (Teeri et al., 2006). Furthermore, the high variation in flower color and patterning of ray and disc florets as well as the high levels of secondary metabolites derived from connected pathways make it a putative model crop for biosynthetic research (Teeri et al., 2006). Given the importance of gerbera in floriculture and breeding as well as its potential for fundamental research on flower developmental and regulation of secondary metabolites, there is a demand for genomic resources.

In general, the use of molecular markers in breeding for ornamental crops has been lagging behind other agricultural and horticultural crops (Arens et al., 2012; Smulders et al., 2012). This is partly due to some breeding traits for ornamentals like flower color that are themselves easily visible markers. Also, it is more complicated to develop molecular markers for ornamental crops since they are highly heterozygous with complex genetic background (Debener, 2009). In gerbera, there are only a small amount of SSR and RGA (resistance gene analog) markers (Gong and Deng, 2010, 2012; Seo et al., 2012) available for genetic studies in this species.

With the rapid progress in high-throughput next-generation sequencing (NGS) technologies, new possibilities for creating genomic resources and identifying (SNP) markers have become feasible. Transcriptome RNA sequencing (RNA-seq) provides significant advantages for ornamental crops where genomic resources are still scarce and high levels of heterozygosity are expected. Because gerbera has a relatively large genome size, sequencing transcripts as a genome complexity reduction not only reduces cost and time significantly, but also contributes to establishment of resources by the focus on genes. Furthermore, in species with a very high diversity, many SNPs may not be useable markers because of flanking SNPs. Targeting genic regions which have a lower expected SNP diversity may reduce this and result in more widely applicable markers. At present only two studies have contributed to genomic resources building in gerbera. Using Sanger sequencing, an ESTs database with nearly 17,000 cDNA sequences was already constructed for mining genes involved in gerbera floral development (Laitinen et al., 2005). A transcriptome of the gerbera ray floret sequenced by NGS sequencing was constructed to predict genes involved in gibberellin metabolism and signal transduction (Kuang et al., 2013). Although, these transcriptome analyses have been reported in gerbera, these studies were not focussed on finding SNP markers and focussed strictly on flowers.

SNPs that can be discovered from expressed sequence tags (ESTs) NGS-sequencing are valuable resources for genetic research and accepted as markers for MAS in ornamentals (Shahin et al., 2012; Koning-Boucoiran et al., 2015). RNA-Seq can generate numerous transcripts with sufficient read-depth to guarantee high quality SNP identification (Kim et al., 2014). Development of SNP markers for the highly heterozygous ornamentals is very feasible and 200–1000 SNP markers will be sufficient to construct a genetic map for QTL mapping (Smulders et al., 2012).

In this study, we aim for the identification of SNP markers from the transcriptomes of four gerbera genotypes based on leaf and flower tissues using NGS sequencing. Through alignment of reads from four genotypes with consensus contigs constructed by *de novo* assembly, we expect to identify SNPs within and between cultivars and detect reliable SNPs markers that can be used for mapping and other genetic studies. Transcriptomes are analyzed by gene annotation and predicted candidate genes that relate to disease resistance pathways, and to gerbera gray mold in particular, will be shown as examples. Gerbera gray mold is a main problem in gerbera production in greenhouses which is caused by *Botrytis cinerea*. As a necrotrophic pathogen, a series of plant secondary metabolites from the phenylpropanoid and flavonoid biosynthesis pathway are considered to be involved in plant defense responses (Dixon, 2001; Dixon et al., 2002). Phytohormone jasmonate (JA) and ethylene (ET) also play a role in plant defense against *B. cinerea* (Thomma et al., 2001). Identifying the gene sequences involved in these pathways will help us to study their gene function in gerbera upon Botrytis infestation. SNPs found will provide genetic tools for gerbera breeding that may help in efficient gerbera improvement.

## Materials and methods

### Plant materials, RNA isolation and cDNA library construction

Three Mini Gerbera breeding lines (“SP1,” “SP2,” “FP1”) and a garden gerbera breeding line (“FP2”) that are also the parents of two gerbera populations were used for cDNA sequencing. The selected 4 parental genotypes show different symptoms on Botrytis susceptibility and the two populations of these parents showed the largest variation on Botrytis susceptibility among 20 populations tested. Young leaves and floral buds of the four parents were collected and stored at −80°C upon RNA isolation.

Total RNA of the leaves and floral buds for the four parents was isolated according to the standard TRIZol reagent protocol (Life Technologies, USA) followed by purification using the RNeasy isolation Kit (Qiagen, Germany). Total RNA of leaves and floral buds was mixed in equal amounts and sent to GATC Biotech (Germany) for sequence library preparation.

### Sequencing, assembly and SNP detection

The cDNA libraries of all four genotypes were sequenced using 2 × 100 bp paired-end sequencing on an Illumina HiSeq platform (Illumina, USA). Reads were pre-processed using ConDeTri (Content Dependent Read Trimmer) (Smeds and Künstner, 2011) with default settings to trim adapter sequences from the 3′ and 5′ ends from reads and to filter reads with low quality. To improve the quality of assemblies, FLASH (Fast Length Adjustment of Short reads) (Magoč and Salzberg, 2011) was used with default settings to merge overlapping read pairs. For *de novo* assembly, transcripts of four parents were constructed separately by Trinity (Grabherr et al., 2011) from the merged, single-end and paired-end reads.

Construction of a reference transcriptome was performed in a stepwise procedure. In short, transcriptome of SP1 was assembled *de novo* and redundancy was removed by reassembling the transcriptome using CAP3 (Huang and Madan, 1999) with default setting and an identity (−p) of 95%. Next, the transcripts of SP2 were added to the CAP3 contigs and singlets of SP1 and assembled again with the same settings. In a similar way the transcripts of FP1 and FP2 were added and contigs were reassembled. The final consensus contig sequences were used as a reference transcriptome for SNP detection.

For SNP detection the raw reads were pre-processed using Prinseq-lite (vs. 0.20.3) which included the trimming of nucleotides having a phred score lower than 25, the trimming of poly A/T tails, the removal of duplicate reads, of low complexity reads (DUST approach), of reads shorter than 50 bp and of reads with more than one ambiguous nucleotide. The remaining reads of each genotype where aligned to the reference transcriptome using Bowtie2 (–very-sensitive setting) and filtered for mapping quality (>2) using SAMtools (Li et al., 2009). The resulting sam files were merged and used for SNP calling using QualitySNPng (Nijveen et al., 2013) with default settings. Retrieved SNP regions were blasted (BLASTn, *e*-value: 1E-30) to the contigs derived from the EST sequences as a control for possible paralog presence.

### GO annotation, enzyme code annotation and KEGG annotation

To predict function, assembled unigene contigs were annotated. Gene ontology (GO) annotation in Blast2GO (Conesa et al., 2005) consisted of three steps: blasting, mapping and annotation. The assembled contigs were compared by BLASTX against the NCBI non-redundant protein database (nr) using Blast2GO V.3.0. The expectation value (*E*-value) threshold was set at 1E-3 for reporting matches and the number of retrieved hits at 20 (default value). After blasting, Gene Ontology (GO) terms associated to the hits were mapped. When a BLAST result is successfully mapped to one or several GO terms, GO annotations were assigned.

Enzyme code (EC) annotation was available only for contig sequences with GO annotations with EC numbers. Additionally, the KEGG mapping was done to display enzymatic functions in the context of the metabolic pathways in which they participate.

## Results

### Sequencing and SNP detection

The transcriptome reads of four genotypes were obtained using Illumina 2 × 100 bp paired-end sequencing. For genotype “SP1,” sequencing the cDNA library resulted in a total of 114,519,206 raw paired end reads. After trimming and removing reads with low quality 80,182,250 (70%) paired end reads remained. Merging connected paired-end reads using FLASH software resulted in 46,043,245 single reads and 5,931,379 paired end reads for *de novo* assembly. The *de novo* assembly for “SP1” resulted into 113,970 transcripts longer than 200 bp. Results of sequencing assembly data for all four genotypes are shown in Table 1. All raw data has been donated to the SRA (Short Read Archive) and can be found under accession numbers PRJEB12127.

**Table 1:** **Summary of the sequence assembly data**.

**Parents**	**# bases**	**# paired end reads**	**Pre-processing**			***De novo* assembly**	**CAP3 Assembly**	
			**# paired end reads after trimming**	**# paired end reads after merging**	**# single end reads after merging**	**# transcripts (>200 bp)**	**# contigs**	**# singlets**
SP1	11,566,439,806	114,519,206	80,182,250	5,931,379	46,043,245	113,970	36,770	144,356
SP2	11,885,597,584	117,679,184	82,276,886	6,964,989	46,331,282	119,675		
FP1	14,435,581,146	142,926,546	99,901,704	9,400,798	55,225,869	130,234		
FP2	9,416,840,444	93,236,044	73,156,762	6,104,039	38,065,122	73,634		

**Figure 1:**
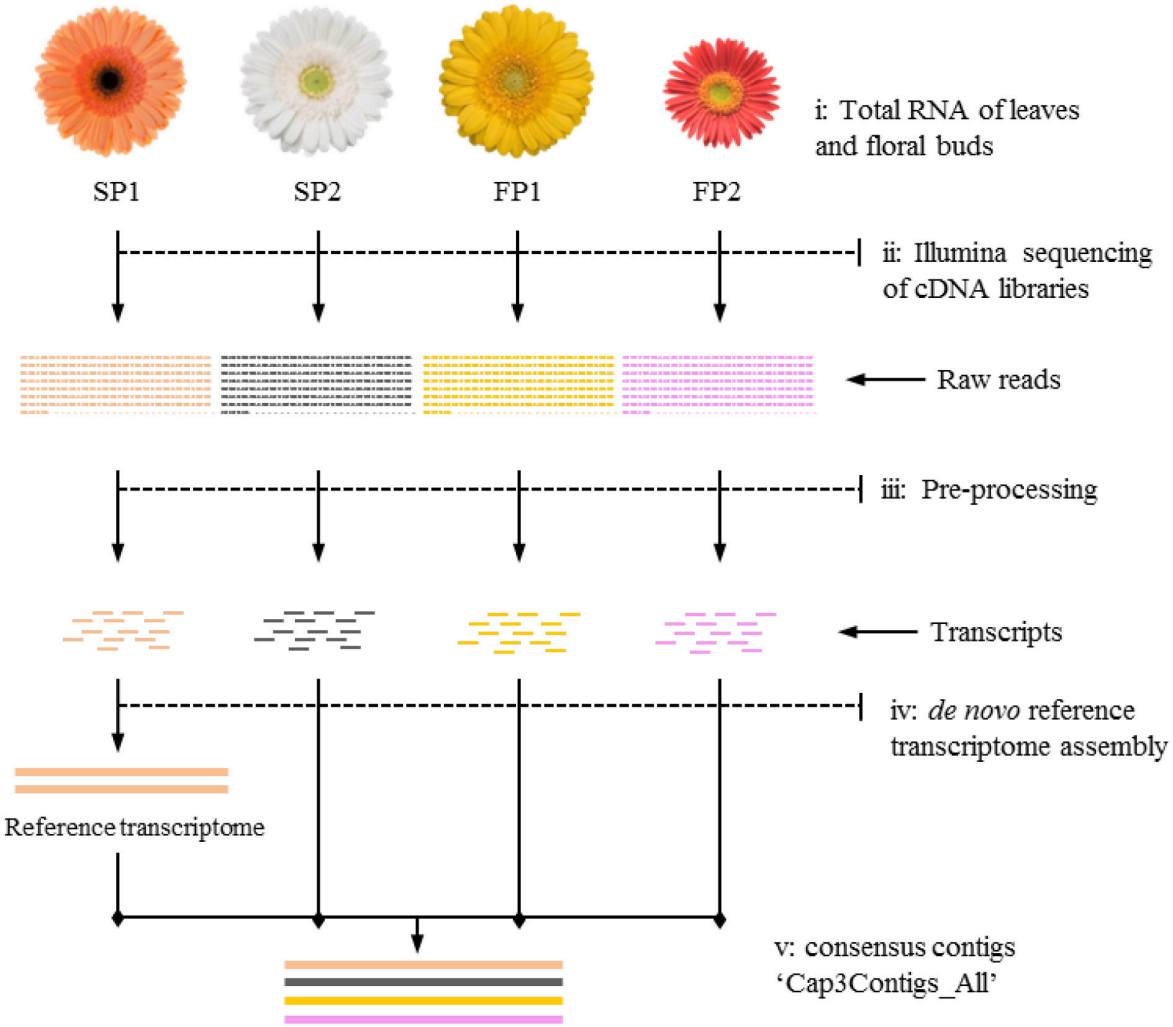
**Workflow of transcriptome sequencing for four parents**. **(i)** Leaves and floral buds of the four gerbera genotypes used to isolate RNA; **(ii)** Mixed cDNA libraries of four genotypes sequenced on an Illumina platform; **(iii)** Raw reads used for pre-processing to trim adapter sequences and to filter reads with low quality; **(iv)** Transcripts of SP1 assembled as first step toward reference transcriptome construction; **(v)** Transcripts of other genotypes mapped to reference to yield consensus contigs named “Cap3Contigs_All” for SNP detection and annotation.

Transcripts of parent “SP1” were first used to construct a reference transcriptome after which transcripts of the other genotypes were one by one added to reach an overall consensus assembly (Figure 1). The final consensus transcriptome yielded 36,770 consensus contigs and 144,356 singletons. The average length of consensus contigs was 1397 bp, and the N50 was equal to 1889 bp (The minimum length of 201 bp, median length of 1130 bp and maximum length of 15746 bp). This consensus transcriptome (named “Cap3Contigs_All”) was the starting point for SNP identification and annotation. All paired-end and single-end reads of the four genotypes were mapped to the 36,770 “Cap3Contigs_All” consensus contigs for SNP detection. In total 398,917 SNPs polymorphic within or between the four genotypes were detected. Genetic diversity of the consensus sequences on average is 7.8 SNPs per kb. The average SNP density within the four genotypes varied from 3.7 to 4.8 SNPs per kb of sequence. They all harbor quite a lot parent specific SNPs and population specific SNPs polymorphic in only that specific parent or population (Table 2).

**Table 2:** **SNPs on parents and consensus sequences**.

**Population**	**Parents**	**#SNPs present in parent^a^**	**SNPs per kb^b^**	**#SNP genotype specific^c^**	**#SNP population specific^d^**	**Total polymorphic SNPs^e^**	**Average polymorphic position per kb^f^**
S	SP1	218,049	4.2	16,490	19,762	398,917	7.8
	SP2	220,590	4.3	20,852			
F	FP1	245,063	4.8	27,780	17,189		
	FP2	190,047	3.7	23,741			

a *Total number of SNPs present in each parental genotype*.

b *SNPs per kb of sequenced data in four parental genotype*.

c *Number of unique SNPs in each parental genotype*.

d *Number of unique SNPs in each population*.

e *Total number of SNPs within and between the four parental genotypes in the 36,770 consensus contigs*.

f *Average polymorphic sites per kb of the four parental genotypes combined*.

### GO, EC, KEGG annotation

The 36,770 consensus contigs “Cap3Contigs_All” were used for blasting against the NCBI non-redundant protein database (nr) to look for the most similar proteins for each contig (Table 3). A total number of 29,146 contigs gave BLAST hits (79.2%). Out of the best-hit for every contig with BLAST result (See Table S1), the *E*-value of 9398 contigs (~32%) is below an value of 1e^−180^, and 2481 contigs (8.5%) with the *E*-value greater than 1e^−20^, others are in between. Sequences similarity distribution chart (see Figure S1A) shows that most (91.2%) of the BLAST hits have sequence similarity values higher than 60% and half of them (50.4%) higher than 80% with our gerbera consensus contigs. Most blast hits were found from grape (*Vitis vinifera*), soybean (*Glycine max*), poplar (*Populus trichocarpa*), potato (*Solanum tuberosum*), tomato (*S. lycopersicum*) and cacao tree (*Theobroma cacao*) (Figure S1B). Most of these species also feature in the top hits distribution with grape as main contributor (Figure S1C).

**Table 3:** **Transcriptomes annotation process results**.

**Annotation step**	**Contig no**.
Contigs without blast^a^	55
Contigs blasted without blast hit	7569
Contigs with Blast hit, without GO mapping	3018
Contigs with GO Mapping, without GO annotation	4771
Contigs B2G Annotated	21,357
Total contigs	36,770

a *Contig sequence longer than 8000bp*.

The GO terms were obtained during the mapping step. Out of sequences with blast results, 73.3% (21,357 contigs) could be GO annotated. The sequences with GO annotation were described in terms of biological processes, cellular components and molecular functions. Top 20 GO terms of the three separate aspects were listed in Table S2.

Enzyme annotations were also done in contigs with GO annotations. A total of 8761 contigs eventually showed EC numbers and the enzyme code distribution is shown in Table 4. KEGG mapping displayed enzymatic functions in the context of the metabolic pathways in which they participate. The EC annotated contigs are involved in a total of 144 different metabolic pathways, including all kinds of carbohydrate metabolic pathways, amino acid metabolic pathways, nitrogen metabolic pathways, as well as a series of secondary metabolic biosynthesis pathways. The top 30 pathways in overall sequence coverage and the details of all 144 pathways with contig identity and enzyme code can be found in Tables S3, S4, respectively.

**Table 4:** **Gerbera transcripts enzyme code distribution**.

**EC Classes**	**#Contigs**
1. Oxidoreductases	1769
2. Transferases	3404
3. Hydrolases	2403
4. Lyases	421
5. Isomerases	281
6. Ligases	483

### Transcripts related to phenylpropanoid biosynthesis and flavonoid biosynthesis pathway

**Figure 2:**
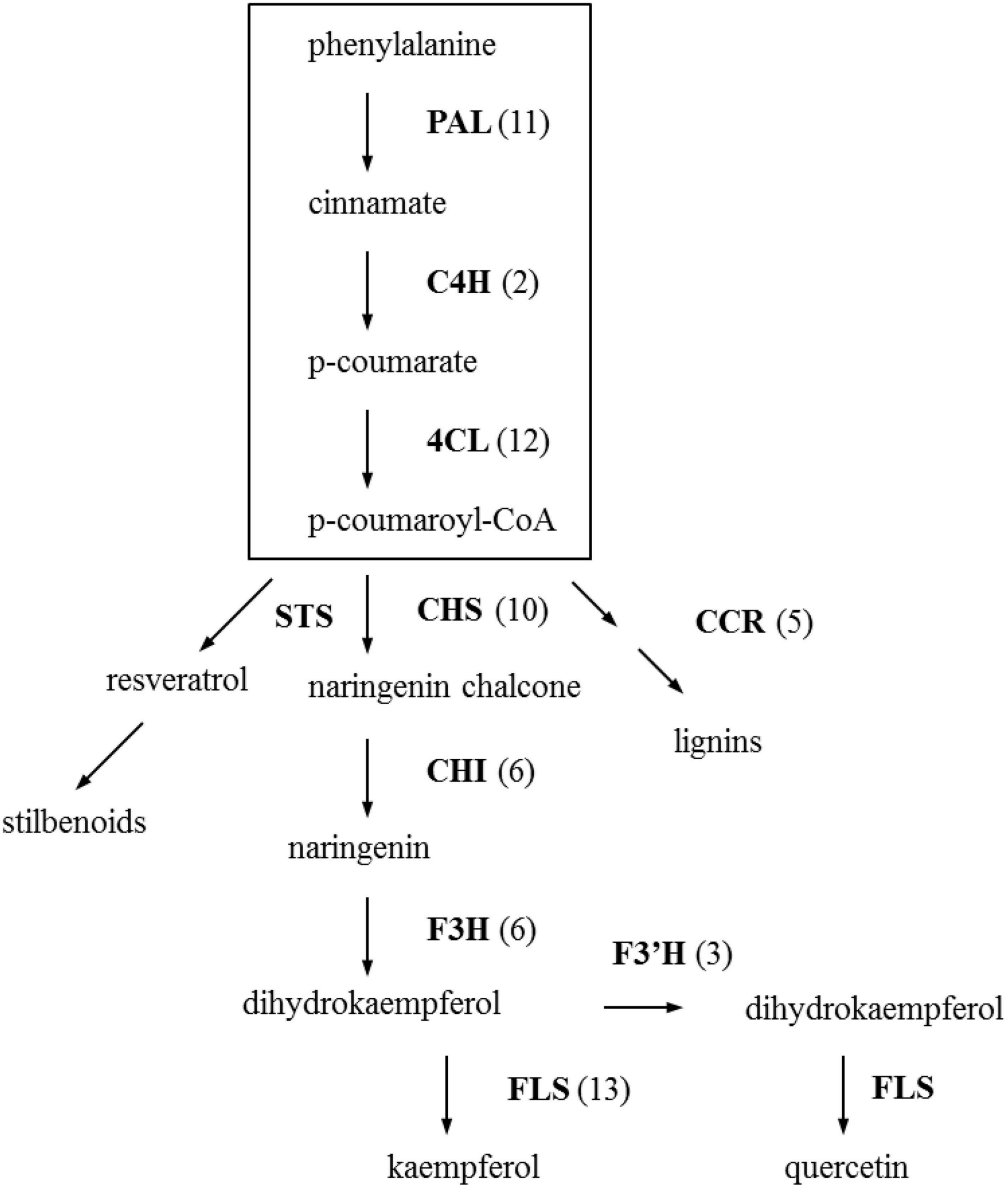
**Distribution of Gerbera transcripts in the simplified phenylpropanoid and flavonoid biosynthesis pathway (Dixon et al., 2002; Ainasoja, 2008; Ali et al., 2011; Ferreyra et al., 2012; Boubakri et al., 2013)**. The processes in the box indicate the general phenylpropanoid pathway and the rest is flavonoid pathway. Each enzyme name is followed with the number of contigs homologous to the gene family encoding this enzyme between brackets. PAL, phenylalanine ammonia-lyase; C4H, cinnamate-4-hydroxylase; 4CL, 4-coumarate-CoA ligase; CHS, chalcone synthase; STS, stilbenes synthase; CCR, cinnamoyl-CoA reductase; CHI, chalcone isomerase/chalcone-flavanone isomerase; F3H, flavanone 3-hydroxylase; F3′H, flavonoid 3′-hydroxylase; FLS, flavonol synthase.

Based on the EC annotated sequences, enzymes involved in phenylpropanoid and flavonoid biosynthesis pathway that are considered to be involved in flower color and disease resistance were retrieved and highlighted in different colors in the pathway-maps from KEGG (see Figures S2, S3). There are 137 contigs that translate to 14 enzymes in the phenylpropanoid biosynthesis pathway. Eleven enzymes represented by 71 contigs were found for the flavonoid biosynthesis pathway. These two pathway-maps loaded from KEGG (see Figures S2, S3) included all possible enzymes and metabolites in a broad perspective, but we can see clearly from the simplified phenylpropanoid and flavonoid biosynthetic pathway (Figure 2) that the key enzymes in the pathways are well represented. The three key regulatory enzymes in the phenylpropanoid biosynthesis are phenylalanine ammonia lyase (PAL, EC:4.3.1.24), cinnamate-4-hydroxylase (C4H, EC:1.14.13.11), 4-coumarate-CoA ligase (4CL, EC:6.2.1.12), which were represented by 11, 2, and 12 homologous contigs respectively. The sequence similarities of the best-hit for these 25 contigs are above 90% and 17 of the best-hits come from other species within the Compositae like, *Helianthus tuberosus, Lactuca sativa, Artemisia sieberi, Cynara cardunculus*, etc indication that these pathways are well conserved within the family. After the formation of p-coumaroyl-CoA, the next step is into the central flavonoid pathway. Chalcone synthase (CHS, EC:2.3.1.74) and chalcone isomerase (chalcone-flavanone isomerase, CHI, EC:5.5.1.6.) are the first two enzymes in the flavonoid pathway leading to subsequent metabolite synthesis. Eight out of the 10 contigs which are predicted as *CHS* gene are highly identical to the gerbera *CHS* genes in public databases (Z38096.1, Z38097.2, Z38098.1, AM906210.1, AM906211.1, X91339.1) with a very low (or zero) *E*-value and similarity close to 100%. No contig were found specific for stilbene synthase (STS, EC:2.3.1.95) which is the key enzyme for stilbene synthesis. Enzymes in this pathway and the number of contigs homologous to these enzymes are show in Figure 2.

### Transcripts related to phytohormone biosynthesis and signaling

**Figure 3:**
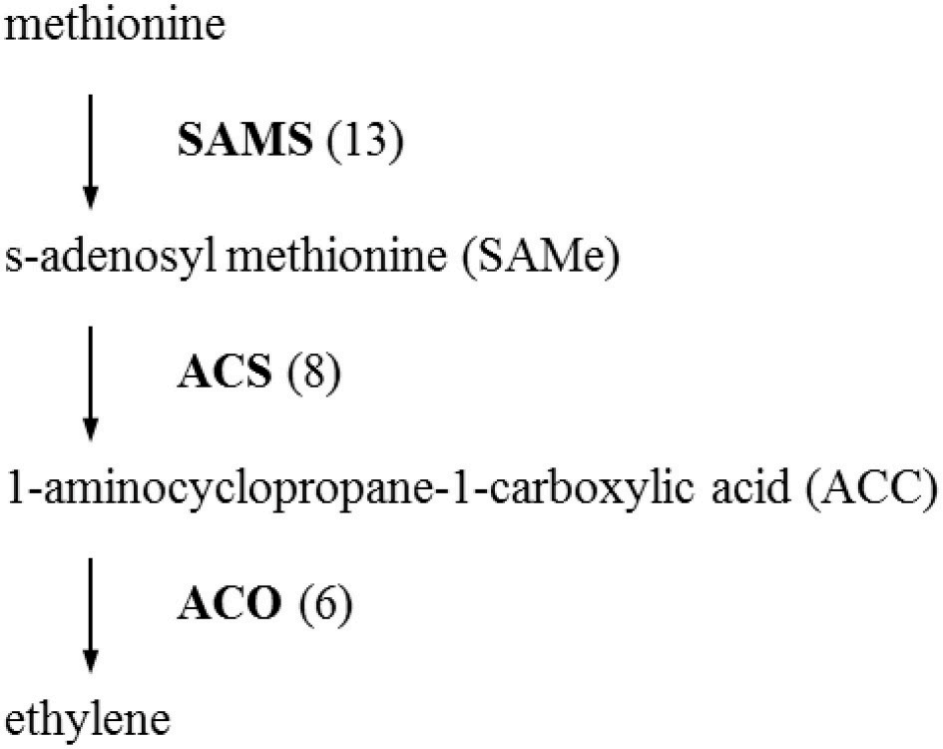
**Distribution of Gerbera transcripts in the ethylene biosynthetic pathway (Wang et al., 2002)**. Each enzyme name is followed with the number of contigs homologous to the gene family encoding this enzyme between brackets. SAMS, S-adenosyl methionine synthase; ACS, 1-aminocyclopropane-1-carboxylic acid (ACC) synthase; ACO, ACC oxidase.

**Figure 4:**
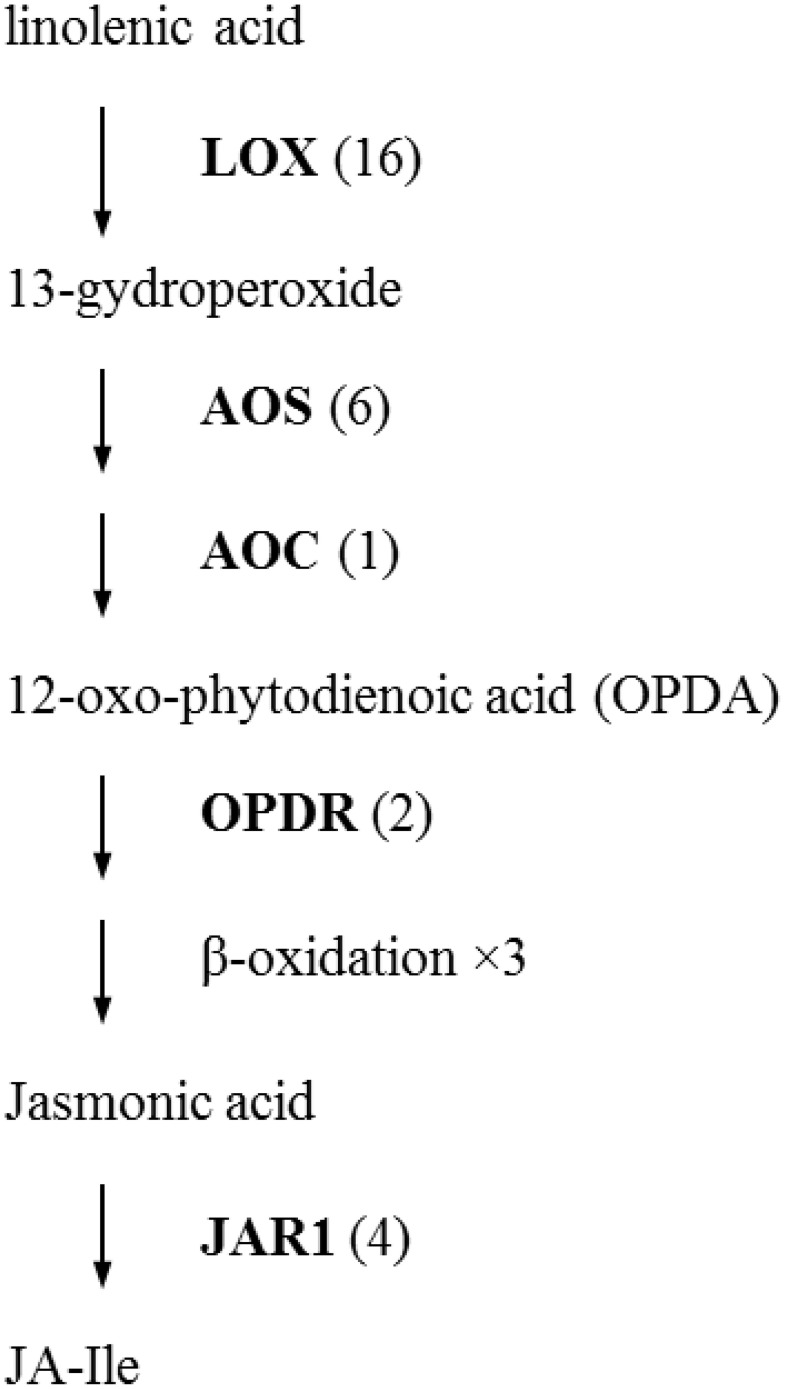
**Distribution of Gerbera transcripts in the Jasmonic acid biosynthetic pathway (Howe, 2001; Wasternack, 2007)**. Each enzyme name is followed with the number of contigs homologous to the gene family encoding this enzyme between brackets. LOX, lipoxygenase; AOS, allene oxide synthase; AOC, allene oxide cyclase; OPDR, 12-oxo-phytodienoic acid reductase; JAR1, JA amino acid conjugate synthase; JA-Ile, jasmonate-isoleucine.

**Figure 5:**
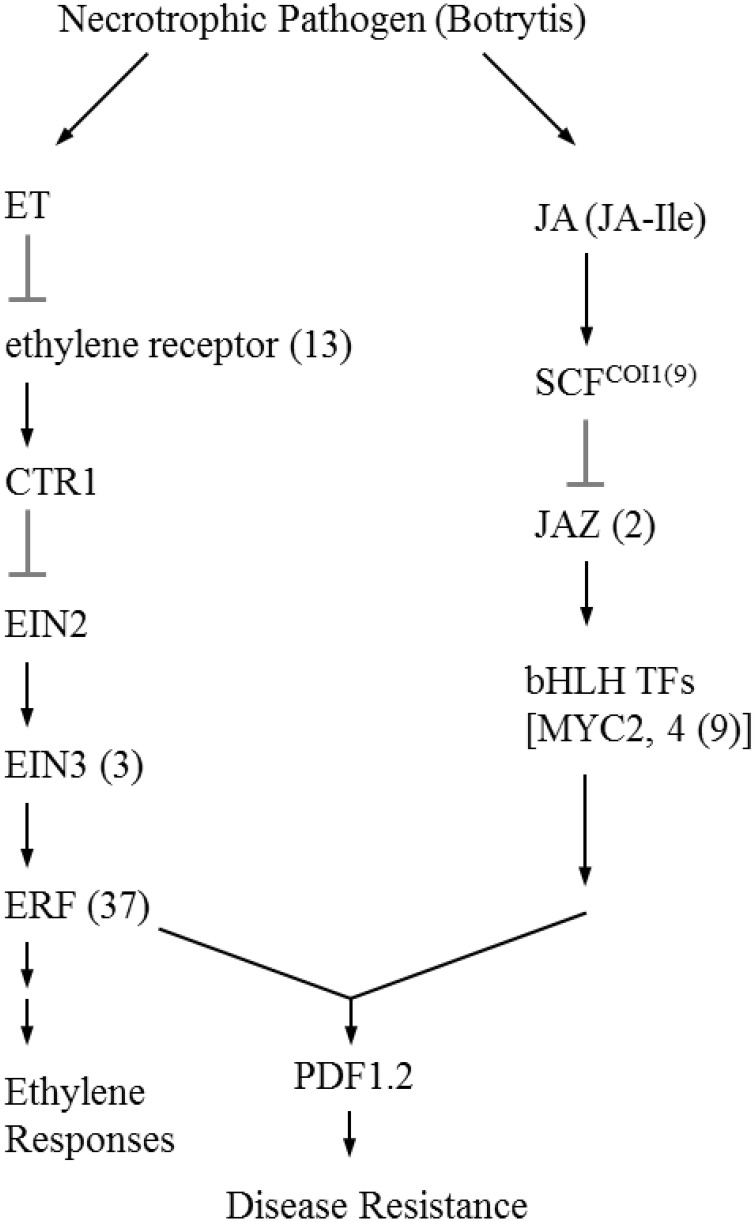
**Simplified signaling ethylene and jasmonic acid signal transduction during Botrytis infection (Wang et al., 2002; Katsir et al., 2008a)**. Each enzyme name is followed with the number of contigs homologous to the gene family encoding this enzyme between brackets. ET, ethylene; CTR1, constitutive triple response 1; EIN2, ethylene insensitive 2; EIN3, Ethylene insensitive 3; ERF; ethylene response factor; JA, jasmonate; JA-Ile, jasmonate-isoleucine; SCF, Skp/Cullin/F-box; COI1, coronatine-insensitive 1; JAZ, jasmonate ZIM domain; bHLH TFs, basic-helix-loop-helix transcription factors; PDF1.2, plant defensin 1.2.

The initial precursor for ethylene synthesis is the amino acid methionine. The three key regulatory enzymes in the pathway are S-adenosyl-l-methionine synthase (SAMS, EC:2.5.1.6), ACC synthase (ACS, EC:4.4.1.14) and ACC oxidase (AOC, EC:1.14.17.4). Our gerbera EST database contains multiple contigs encoding these three enzymes (see Figure 3). Jasmonate biosynthesis start from linolenic acid. Lipoxygenase (LOX, EC:1.13.11.12), allene oxide synthase (AOS, EC:4.2.1.92), allene oxide cyclase (AOC, EC:5.3.99.6) and 12-oxo-phytodienoic acid reductase (OPDR, EC:1.3.1.42) participant in the synthesis. Numbers of contigs encoding these enzymes are show in Figure 4. We also found the multiple contigs connected with these two plant hormone signaling pathway which were shown on Figure 5, although some of them still remained without coverage.

## Discussion

In this study, we developed transcriptomes (based on leaf and flower tissues) of gerbera using RNA sequencing in four gerbera genotypes. After *de novo* assembly, we generated 36,770 consensus contigs with an average length of 1397 bp and a N50 length of 1889 bp. Average length and N50 length are markedly larger than a transcriptome of flower development (845 bp and 1321 bp respectively; Kuang et al., 2013). This is likely because four gerbera genotypes were used instead of one which increases chances for higher read coverage and wider sequence overlap.

SNPs present within genotypes and between genotypes were detected from the alignment of reads of all parents with the consensus contigs. In all those consensus contigs with a total length of 51,360,054 bp, 398,917 polymorphic loci (SNPs present either in parent or between parents) were identified with an average SNP density of 7.8 SNPs per 1kb whereas within genotype SNP density ranged from 3.7 to 4.8 SNPs per 1 kb. These numbers are comparable to for instance rose 4–6 SNP/kb (Koning-Boucoiran et al., 2015) and sunflower 6.1 SNP/kb (Bachlava et al., 2012). In eleven safflower (*Carthamus tinctorius*) individuals exons and introns sequences of 7 genes showed a SNP density of 10.5 SNP/kb (Chapman and Burke, 2007) whereas SNP densities varied from 2.6 to 26.9 SNP/kb in 10 intron regions from eight safflower accessions (Chapman et al., 2007). The frequency of polymorphisms in safflower seems higher than that in overall consensus gene sequences of gerbera but this could be biased because of the small set of genes studied in safflower. Furthermore, polymorphism rates in intron are higher than in exons since introns are under less strict selection pressure (Tamura et al., 2013).

The highest numbers of homologs were found with *Vitis vinifera* (grapevine). Interestingly, grapevine is a crop that also is known for its high number of secondary metabolites and its interaction with *B. cinerea*. Many studies have been performed on this crop-pathogen from multiple aspects (Jeandet et al., 1991; Hain et al., 1993; Goetz et al., 1999; Coutos-Thévenot et al., 2001; Bézier et al., 2002; Poinssot et al., 2003; Trotel-Aziz et al., 2006; Deytieux-Belleau et al., 2009; Timperio et al., 2012) that could be instructive for the interaction of the pathogen with gerbera as well.

There is general recognition that various natural secondary metabolites play an important role in plant defense (Dixon, 2001; Dixon et al., 2002; Howlett, 2006; van Baarlen et al., 2007). Plants combating the necrotrophic pathogen *Botrytis* especially utilize this tool (Oliver and Ipcho, 2004; Glazebrook, 2005). The precursors for these compounds, which are involved in physical and chemical barriers, such as lignin and phytoalexins, are derived from the phenylpropanoid pathway. Enzymes involved in the biosynthesis of the general phenylpropanoid pathway have been well studied (see Dixon et al., 2002). For instance, enzyme activities increased lignification in wheat upon *B. cinerea* infection (Maule and Ride, 1976, 1983). In our study, we identified homologs for the three key genes (PAL, C4H, and 4CL) in the core of the phenylpropanoid pathway in gerbera, and found multiple transcripts encoding the three enzymes. For 17 out of the 25 best-hits homologs come from *Compositae* species.

The flavonoid pathway is closely linked with the phenylpropanoid pathway and the precursor is a phenylpropanoid-derived compound. Flavonoid biosynthesis will yield in different flower color pigments (Winkel-Shirley, 2001) but may also produce a range of plant defense compounds (Treutter, 2005). For instance, chalcone synthase (CHS) belongs to the type III polyketide synthase (PKS) superfamily (Austin and Noel, 2003; Abe and Morita, 2010) and is the key enzyme toward the flavonoid biosynthesis. Members in the type III PKS superfamily, including stilbene synthases (STS) and 2-pyrone synthase (2-PS) in gerbera, share high amino acid similarities and are highly correlated with Botrytis resistance. Grapevine synthesizes stilbenes upon Botrytis infection (Jeandet et al., 1991; Goetz et al., 1999). Tobacco transformed with a stilbene synthase gene from grapevine showed increased resistance to *B. cinerea* (Hain et al., 1993). The 2-pyrone synthase (2-PS) codes for polyketide synthase which synthesizes a putative precursor for two phytoalexins in gerbera. Knocking out this gene resulted in increased susceptibility to *B. cinerea* (Koskela et al., 2011). Deng et al. (2014) confirmed that CHS enzymes in gerbera are encoded by a family of three genes. We found at least 10 transcripts annotated to the chalcone and stilbene synthase family protein. Eight of them showed high similarity at nucleotide level with gerbera *CHS* or *CHS*-like genes in public databases, whereas the other two showed only low amino acid similarity (40%) to known gerbera sequences. The latter two give the best hits to chalcone and stilbene synthases from *T. cacao* (XP_007041944.1, 90%) and a putative chalcone synthase from *Artemisia annua* (ACY74337.1, 93%). Based on the phylogenetic tree of the amino acid sequences of chalcone- and stilbene-like synthases (Figure S4 and Table S5), these two transcripts (GhCHS1 and GhCHS2 in Figure S4) belong to a clearly separate group of putative chalcone and stilbene synthases.

In this study, we also exampled the possibilities of the presented transcriptome on plant hormone ethylene and jasmonate biosynthesis and signaling networks that are considered to play an important role in plant resistance in general and Botrytis in specific. For instance, ethylene production plays an important role in plant resistance against *B. cinerea* (Broekaert et al., 2006). The rate-limiting step of ethylene synthesis is the conversion of *S*AMe to ACC by ACC synthase (ACS; Kende, 1993). A multigene family codes for ACC synthase in plants. Nine contigs were found in our data related with ACS genes. Similarly, the Arabidopsis and tomato genomes contain nine ACS genes (Harpaz-Saad et al., 2012). Two knockout mutants of type I ACS isoforms in Arabidopsis, *acs2* and *acs6*, reduced *B. cinerea*-induced ethylene biosynthesis (Han et al., 2010). ACS-silenced apple fruit was more susceptible to *B. cinerea* than untransformed apple (Akagi et al., 2011). The activity of lipoxygenase (LOX), a key JA biosynthetic enzyme, is also highly related to Botrytis resistance (Azami-Sardooei et al., 2010). Our gerbera EST database contained multiple transcripts encoding key enzymes in ethylene and jasmonate synthesis pathways. An efficient defense response to Botrytis also need genes in signaling transduction pathways, such as EIN2 in ethylene signaling (Thomma et al., 1999), COI1 and JAZ in jasmonate signaling (Cerrudo et al., 2012). COI1 protein was shown to mediate JAZ degradation to release its bound downstream TFs (e.g., MYC2) for defense gene expression (Katsir et al., 2008b; Kazan and Manners, 2013). Jasmonoyl–isoleucine (JA-Ile), which is the only bioactive jasmonates derivative by a JA conjugate synthase (JAR1) confirmed so far, directly promotes their interaction (Katsir et al., 2008a; Wasternack, 2007). In Arabidopsis, the *coi1* and other mutations that block functional JA signaling, showed increased susceptibility to Botrytis and decreased induction of the plant antimicrobial metabolite camalexin after infection (Rowe et al., 2010). Some of these components in the signal transduction pathway still remained without coverage which may be related to the RNA-seq source that is from unchallenged material as the main focus was building generic genomic resources and SNP detection.

Through analysis of the large gerbera EST database that was generated from next-generation sequencing, we identified a series of SNP markers for further linkage mapping and also identified transcripts that might be involved in interesting pathways for both fundamental as well as applied studies as was exampled for Botrytis resistance. We expect these genes can provide genetic resources for studying the mechanism of disease resistance and developing markers for gerbera breeding in the future.

## Author contributions

Conception of the study: YF, PA, JV and RV; Practical design of the study: PA, YF and GE; Development of methodology: YF, GE and PA; Assembly and SNP detection: GE; Annotation of transcripts: YF; Writing of the manuscript: YF, GE, and PA. Manuscript modification and discussions: PA, RV and JV. All authors read and approved the final version of the manuscript.

### Conflict of interest statement

The authors declare that the research was conducted in the absence of any commercial or financial relationships that could be construed as a potential conflict of interest.
