# Supplementary material for: Transcriptome Analysis of Gerbera hybrida Including in silico Confirmation of Defense Genes Found
Source: Front Plant Sci. 2016 Mar 1;7:247. doi: 10.3389/fpls.2016.00247 (PMC4771743; doi:10.3389/fpls.2016.00247)
Supplement: Supplementary file 6 [file Image1.PDF]

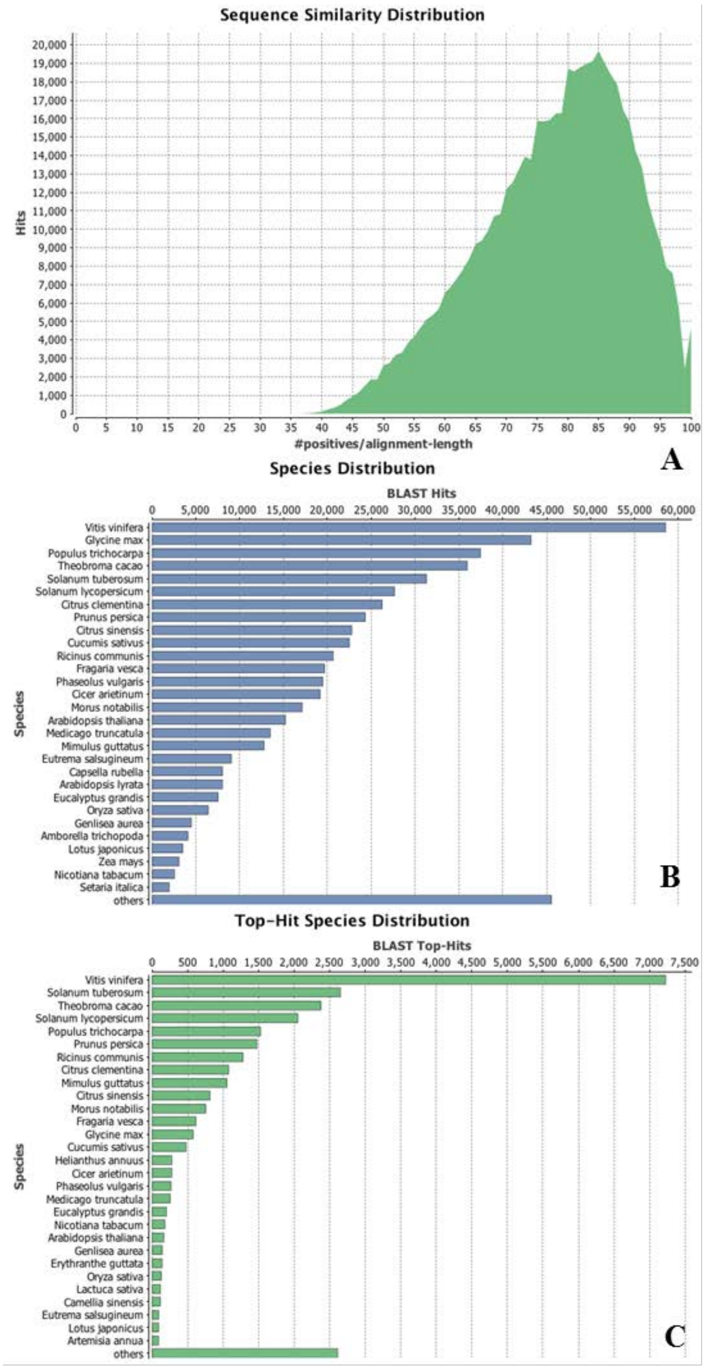

Figure S1 Charts of Sequences Similarity Distribution (A), (Blast hit) Species Distribution (B) and Top-hits Species Distribution (C).
