# Supplementary material for: Transcriptome Analysis of Gerbera hybrida Including in silico Confirmation of Defense Genes Found
Source: Front Plant Sci. 2016 Mar 1;7:247. doi: 10.3389/fpls.2016.00247 (PMC4771743; doi:10.3389/fpls.2016.00247)
Supplement: Supplementary file 9 [file Image4.PDF]

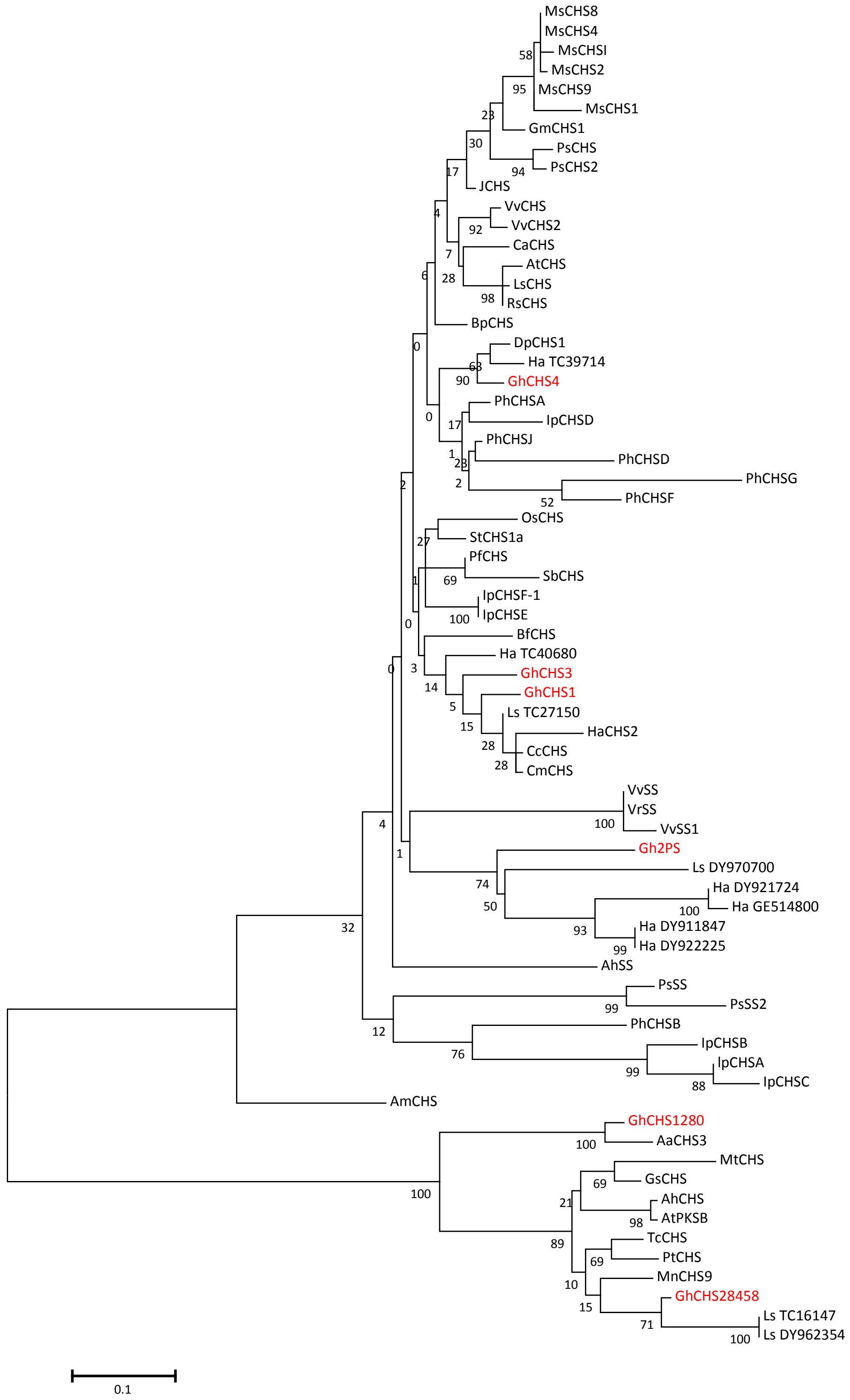

Figure S4 Phylogenetic tree of chalcone and stilbene synthase family protein from different species. The tree generated with MEGA 6.0 using the Maximum-likelihood after sequence alignment. The numbers indicate bootstrap probabilities. NCBI accession numbers of the chalcone and stilbene synthase family protein sequences used in the tree are given in supplementary file Table S5.
