# Supplementary material for: Transcriptome Analysis of Gerbera hybrida Including in silico Confirmation of Defense Genes Found
Source: Front Plant Sci. 2016 Mar 1;7:247. doi: 10.3389/fpls.2016.00247 (PMC4771743; doi:10.3389/fpls.2016.00247)
Supplement: Supplementary file 8 [file Image3.PDF]

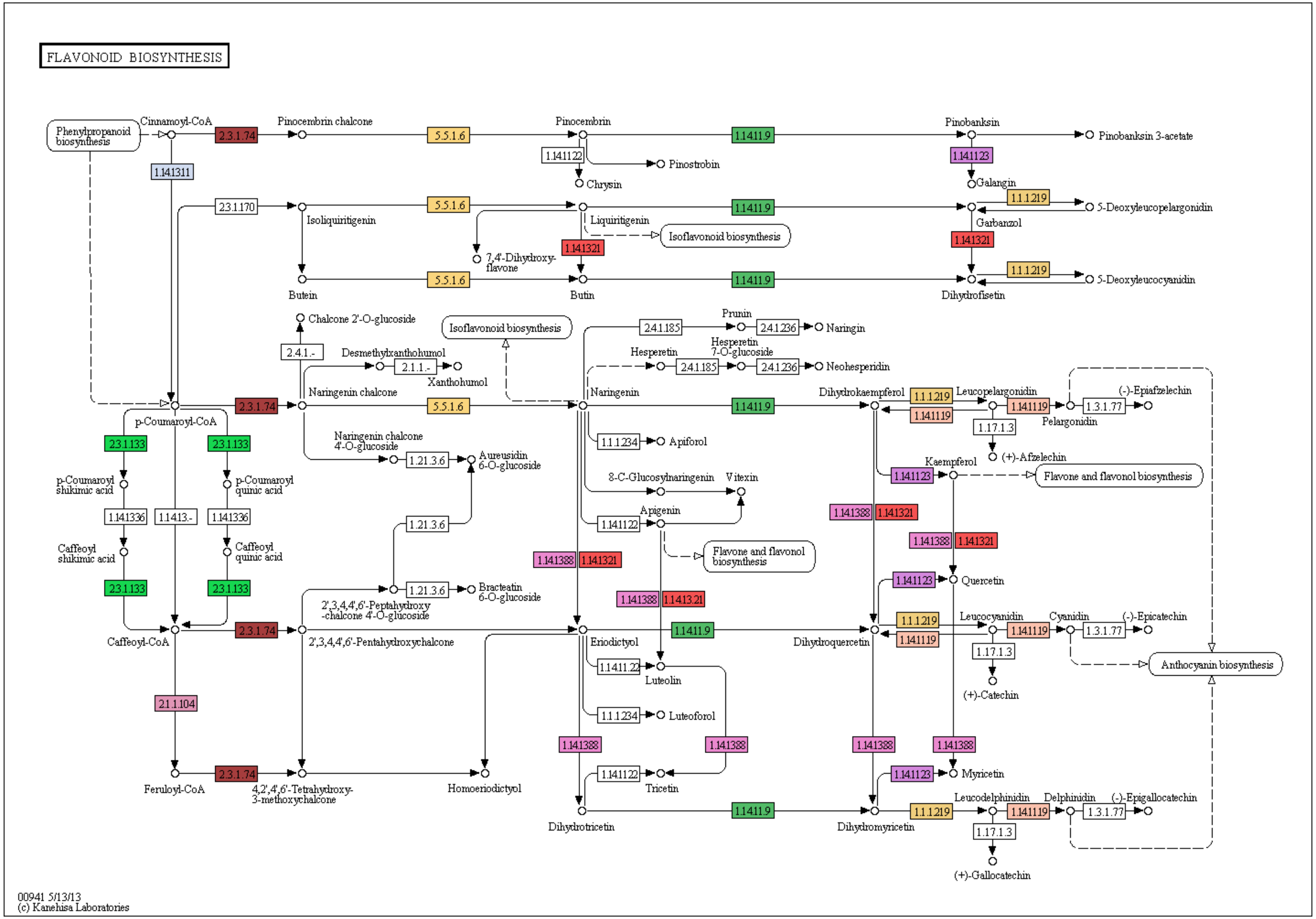

Figure S3 Details of enzymes involved in flavonoid biosynthesis pathway (from KEGG). Annotated enzymes are highlighted in different colors.
